# Do good, stay well. Well-being and work satisfaction among German refugee helpers: A national cross-sectional study

**DOI:** 10.1371/journal.pone.0209697

**Published:** 2018-12-26

**Authors:** Eva Jobst, Christine Gall, Christian Eiche, Torsten Birkholz, Johannes Prottengeier

**Affiliations:** 1 Department of Anesthesiology, University Hospital Erlangen, Erlangen, Germany; 2 Faculty of Medicine, Friedrich-Alexander University Erlangen-Nürnberg, Erlangen, Germany; 3 Department of Medical Informatics, Biometry and Epidemiology, Friedrich-Alexander University, Erlangen-Nürnberg, Erlangen, Germany; Medical University Hospital Tuebingen, GERMANY

## Abstract

**Background:**

Since 2015, more than 3 million refugees have reached the European Union. In order to receive and integrate them, societies heavily rely on relief organizations and private initiatives. Yet the well-being, work-satisfaction and possible health implications for refugee helpers have not been adequately addressed.

**Methods:**

In a German national cross-sectional study, we gathered socio-demographic data on refugee helpers. Work satisfaction was examined by means of Neuberger and Allerbeck’s Work Description Inventory. We screened for depression by using the 5-item WHO Well-Being Index (WHO-5), and for post-traumatic stress disorder (PTSD) using the PTSD Short Screening Scale (PTSD-7). 1712 questionnaires were analyzed.

**Results:**

Females accounted for 73.4% (1235), the mean age was 52.0 years (SD: 14.4). 61.6% were academics (1042). 87.0% (1454) were voluntary helpers who invested 9.4 hours (SD: 8.9) per week. Refugee helpers were more satisfied with the content than with the conditions or the organization of their work. Their work satisfaction and overall life satisfaction reached higher values than in representative samples. The mean WHO-5 index for refugee helpers was 68.2 points (SD: 19.0). Positive depression screening was found in 17.3% (226). 982 (57.4%) had experienced a traumatic event in their past or witnessed it during their work in refugee aid. 33 (2.8%) of the helpers had a positive PTSD screening.

**Conclusions:**

Refugee helpers deliver invaluable services to migrants and receiving communities. Our data indicates above average well-being as well as work-satisfaction. Psychological traumatization is found frequently but fortunately PTSD is rare. All efforts should be made to uphold helpers’ keen spirit and contributions. They should be screened regularly with regards to work satisfaction, well-being and mental health. As part of a comprehensive health promotion strategy they should be instructed about depression, psychological trauma, PTSD and ways to find help.

## Introduction

Since 2015, over 3 million asylum seekers have been registered within the European Union (EU), with over 1 million having been registered in Germany alone. Within the EU, Germany recorded the highest number of applications for asylum in 2015, 2016 and 2017 again [1]. Possibly, the numbers of refugees coming to Europe are yet to increase, for example but not exclusively because of the ongoing war in Syria [2]. Amongst other factors, economic and political reasons have led to increased migration from sub-Saharan Africa nearly every year since 2010 [3]. The arrival of large numbers of refugees can be challenging for the receiving countries [4] and has led to fierce political debate not only in Germany but in the entire European Union [5].

Fortunately, in 2015 many German citizens were eager to help, leading to a great increase of voluntary work in the field of refugee aid over the last few years [6]. Three years after the German “summer of welcome”, relief organizations and private initiatives still dedicate themselves to helping refugees find their way into our society.

Investigations into working-conditions, work-satisfaction, well-being and health threats to refugee helpers are imperative. For this purpose, data must be provided that helps to generate and sustain a favorable environment in which helpers can successfully offer their invaluable services.

But so far, literature mainly provides broader data on the mental health of aid workers deployed abroad, to hostile environments in regions of crisis. Prevalence rates for depression reach up to 68% among international humanitarian staff [7–11] with alarming correlations to the assignment [12].

Moreover, aid workers can experience primary or secondary traumatization during their work [13, 14]. Both forms of traumatization may lead to post-traumatic stress disorder (PTSD) and PTSD-like symptoms [15]. In primary traumatization the victim is directly exposed to a “serious threat” [15]. However, secondary traumatization concerns persons with indirect exposition to a traumatic event such as a victim’s therapist or family member who then develops PTSD symptoms like arousal or avoidance [15]. Thus, secondary traumatization is an adopted traumatization that occurs although the affected person never faced the stressor themselves [16]. This has to be differentiated from McCanns’ and Pearlmans’ “vicarious traumatization” which entails a shift of the therapist’s cognition, self-perception and world views as a consequence of their work with traumatized victims [15, 17]. Apart from that, one might develop what Figley calls “compassion fatigue”: emotional and physical weariness as a consequence of ongoing exposition to “the stress resulting from helping or wanting to help a traumatized person” [15, 18]. Therefore, it is a condition of “burnout” [18]. Personnel working or engaging in refugee aid in Germany might as well be repeatedly exposed to secondary traumatic stress for example when working as interpreters. Kindermann et al. reported PTSD in 9%, subclinical PTSD in 33% and secondary traumatization in 21% of the interpreters working with refugees in Heidelberg [19]. Denkinger et al. demonstrated secondary traumatization in 22.9% of the caregivers who worked in the Baden-Württemberg Humanitarian Admission Program that supports women and children having suffered in the Yazidi genocide in Iraq [20].

Working conditions may also be difficult for refugee helpers being engaged in their home country. The awareness of suffering and possible tensions within the refugee community are only some of the potentially stressful elements refugee helpers are reportedly exposed to [13]. Reports about the living conditions in so-called initial reception facilities in Germany describe a lack of space, hygiene and privacy [21, 22]. These conditions per se might lead to a lower well-being or increased susceptibility for burnout in refugee helpers.

Even though Germany was the main destination of refugees, and the commitment of local helpers was unprecedented, data on refugee helpers, against the background of our high-income country, is very limited. As a consequence, we investigated helpers’ socio-demographics, gathered details about their working-conditions and work-satisfaction, and screened for signs of depression, traumatic events and PTSD.

## Methods

In a nationwide German cross-sectional survey, we collected socio-demographic data on refugee helpers as well as descriptions of their working-conditions, resources and work-satisfaction. We searched for indicators of traumatic events, depression, PTSD and possible risk factors.

The survey was compiled using self-formulated questions alongside several long-established questionnaires: The Work Description Inventory by Neuberger and Allerbeck, the WHO (five) Well-Being Index, and the German version of the Short Screening Scale for DSM-IV Post-traumatic Stress Disorder [23–26].

We used the Work Description Inventory by Neuberger and Allerbeck [24] to obtain details about helpers’ work satisfaction and compare our data with reference values. The inventory consists of 79 items and is divided into seven subscales (my colleagues, my supervisor, my activities, my working conditions, organization and management, my development, my salary), which investigate different aspects of work. A Likert scale with four levels is used to answer the items (1 =“yes”, 2 = “more yes than no”, 3 = “more no than yes”, 4 = “no”). For every subscale, a measure of satisfaction can be calculated which is expressed by a scale mean. In addition, overall life and overall work satisfaction can be determined using Kunin faces ranging from 1 = very dissatisfied (smiley with a downward pointing mouth) to 7 = very satisfied (laughing smiley) [24]. For both measures (scale means and Kunin faces) higher values express higher work satisfaction. Neuberger investigated work satisfaction in different occupational groups to gain reference values [24, 27].

The 5-item World Health Organization Well-Being Index (WHO-5) is a scoring instrument with five items that determines well-being. An overall score from 0 to 100 is determined. Score values of < = 50 were regarded as indicative of depression. A score difference of clinical relevance was defined as > = 10 points [26].

The PTSD Short Screening Scale (PTSD-7) [28] consists of seven items testing for arousal (symptom group D in the DSM IV; two items) and avoidance (symptom group C; five items) [25]. PTSD is a possible diagnosis when at least four out of seven symptoms occur at least 2–4 times a week. The score can range from 0 to 7 [25, 28]. According to the DSM IV symptoms should be present for more than a month [29]. Items in the PTSD-7 refer to the presence of symptoms in the last month [25]. In advance, the candidate is presented a list of traumatic events which can be adapted to suit the context of the investigation [25]. In our case, we adjusted a list containing traumatic events related to refugees [30]. Similar traumatic events were classified resulting in five categories (Table 1). We defined a person as “traumatized” when he/she had experienced at least one traumatic event in the past or witnessed it during his/her work in refugee aid (primary traumatization). Although, the A criterion (stressor criterion) was not eliminated in the DSM-5 [31] there had been an extensive discussion whether there is a requirement for it [32–34]. Because helpers might be distressed by situations not mentioned on the list of potential traumatic events (Table 1), we decided to screen all refugee helpers for PTSD symptoms.

**Table 1:** Shortlist of potential traumatic events*.

Category 1	Shortage of food or water Poor health without access to medical care Homelessness
Category 2	Serious injury as a result of war or imprisonment/ torture Consequences of rape or sexual abuse
Category 3	Suicide Attempted suicide
Category 4	Forced separation of families Children being orphaned
Category 5	Another situation that was very frightening or in which you felt in mortal danger

* Partly adapted from [30].

To collect socio-demographic data we asked about age, gender, whether the refugee help was voluntary or salaried, the weekly pensum of refugee work and the helper’s own migration background. In general accordance with the migration background enquiry ordinance [35], we defined "own migration background" as existent when helpers or their parents were born abroad.

Helpers’ views on available resources were determined using a list of 15 items (e.g. suggesting there should be more financial means for refugee help, helpers’ work should be more acknowledged in political contexts etc.). Agreement was measured on a five-point scale ranging from 1 = strongly disagree to 5 = strongly agree.

From October, 5^th^ until December, 30^th^ 2016 the questionnaire was accessible online by use of the platform SoSci Survey [36]. Invitations to participate in our study were disseminated by e-mail with the help of aid organizations nationwide. We considered the act of participation a sufficient proof of consent to our investigation. Participants could terminate the questionnaire at any time without any negative impact for them. The University of Erlangen-Nuremberg’s research ethics committee waived the need for a formal ethical evaluation of our study, due to the non-interventional, voluntary and anonymized data retrieved from non-dependent individuals, and issued a letter of ethical innocuousness on October the 4^th^ of 2016.

All in all, 1712 data sets were analyzed. Data analysis was conducted in R version 3.4.3 [37] with a confidence interval of 95% and significance level being 5% (p<0.05). To investigate if helpers hold certain risk factors for possible depression or PTSD, linear regression and multivariate logistic regression models were utilized. We therefore assessed characteristics like age, gender, dependence of certain traumas and type of work (volunteer vs. professional). Possible PTSD and depression were used as the dependent variables while helpers’ characteristics were the independent variables.

All data underlying our findings have been provided in a public repository, the Harvard Dataverse (https://doi.org/10.7910/DVN/KWILBZ).

## Results

In our dataset, women accounted for 73.4% (1235) of helpers, the mean age was 52.0 years (SD: 14.4). 11.4% of helpers were working in the medical field, 76.6% of helpers had a non-medical profession (see supporting information for S1 Table). Most refugee helpers were engaged through self-organized initiatives (63.1%) or welfare organizations (17.7%; S2 Table). 87.0% were volunteers (1454) and of those 41.9% (604) were unemployed or inactive at the time of testing. 61.6% (1042) of helpers in the study population held a university degree. At the time of the survey, nearly half of the participants (46.4%) had worked in refugee aid for more than a year and the mean time invested per week was 9.4 hours (SD: 8.9) among voluntary workers. Migration background was found to be present in 175 helpers (10.2%).

Regarding work satisfaction, Table 2 indicates that refugee helpers were more satisfied with their activity itself than with the working conditions or the organization of their work. Scale means were compared to values derived from a representative sample [24, 27] showing that satisfaction in the investigated categories was higher in refugee helpers. The means of the representative sample do not lie in the confidence intervals of the refugee helpers which implies p < 0.05 for a one-sample t-test.

**Table 2:** Work satisfaction expressed by scale means. Refugee helpers’ scale means are compared with those of a representative sample.

Scale	Study cohort				Representative sample
	Mean*		N	Missings	Mean*
	Estimate	95%-CI			
**My activity**	3.44	[3.42; 3.46]	1446	266	3.19
**My working conditions**	2.88	[2.85; 2.91]	1314	398	2.75
**Organization and management**	2.97	[2.93; 3.01]	1178	534	2.84

* Scale from 1 to 4. Higher values express higher satisfaction.

Work satisfaction expressed by means of Kunin scales are shown in Table 3. In relation to reference values from Neuberger [24, 27], work satisfaction concerning the three subscales was higher among refugee helpers. This also applies to overall work and overall life satisfaction. Again, the means of the representative sample do not lie in the confidence intervals of the refugee helpers indicating a p-value < 0.05 (Table 3).

**Table 3:** Work satisfaction expressed by Kunin faces. Refugee helpers’ means are compared with those of a representative sample.

Scale	Study cohort				Representative sample
	Mean*		N	Missings	Mean*
	Estimate	95%CI			
**My activity**	5.81	[5.76; 5.86]	1550	162	5.01
**My working conditions**	5.11	[5.05; 5.18]	1451	261	4.60
**Organization and management**	5.04	[4.96; 5.12]	1341	371	4.38
**Overall work satisfaction**	5.50	[5.44; 5.56]	1366	346	4.74
**Overall life satisfaction**	5.87	[5.81; 5.93]	1305	407	5.00

* Scale from 1 to 7. Higher values express higher satisfaction.

When it comes to possible resources, helpers agreed or strongly agreed that training for critical situations should be provided (89.8%), “Interpreters must be available at all times” (90.0%) and “politicians need to supply more financial means for refugee help” (77.1%; Table 4).

**Table 4:** Resources. Relative frequencies calculated for non-missing answers (N).

Variable	Strongly disagree	Disagree	Undecided	Agree	Strongly agree	N
**Politicians need to supply more financial means for refugee help**	0.8%	5.6%	16.4%	27.9%	49.2%	1314
**Refugee helpers should be trained in dealing with critical situations (e.g. violence, intercultural problems etc.)**	0.3%	2.3%	7.6%	34.4%	55.4%	1320
**Accommodation/initial reception points should be more hygienic**	1.8%	6.3%	21.0%	30.0%	40.9%	1308
**Accommodation/initial reception points should be less noisy**	2.0%	5.5%	23.0%	30.6%	38.9%	1307
**Refugee helpers should be acknowledged more by politicians**	1.4%	4.6%	16.0%	30.3%	47.6%	1318
**Refugee helpers should be acknowledged more by their organization/initiative**	4.2%	12.7%	28.6%	26.3%	28.3%	1295
**Refugee helpers should receive more attention from the media**	3.2%	10.7%	26.9%	29.4%	29.8%	1313
**After key situations helpers should receive more support in the form of supervision, training, days off etc.**	1.0%	3.2%	20.4%	35.3%	40.1%	1307
**Working hours should be flexible when helpers are employed elsewhere**	1.7%	2.4%	26.4%	32.0%	37.6%	1233
**Helpers need more room for privacy in the reception points**	4.6%	7.9%	35.3%	27.2%	25.0%	1258
**Interpreters must be available at all times**	0.5%	2.4%	7.2%	26.0%	64.0%	1314
**Processes need to be improved/ More room for maneuver is needed in my organization**	4.4%	10.6%	31.1%	27.0%	26.9%	1269
**Further education on asylum law/ asylum proceedings is needed**	2.1%	4.7%	19.5%	34.6%	39.1%	1290
**Less time pressure must be sought**	5.7%	10.2%	36.0%	23.9%	24.2%	1261
**Asylum law should be changed and changes should be realized**	0.8%	2.9%	18.4%	23.1%	54.9%	1296

The calculated mean WHO-5 index was 68.2 points (SD: 19.0). Positive depression screening, indicated by a score < = 50, was found in 226 (17.3%) of the participants. In traumatized refugee workers, positive screening for depression was significantly more common (n = 180; 19.1%) when compared to non-traumatized helpers (n = 46; 12.6%; p = 0.0075). In the linear regression model factors like gender, age, being a volunteer and being traumatized had a significant influence on the WHO-5 index. This is shown in Table 5.

**Table 5:** Influence on WHO-5 index by refugee helpers’ characteristics in the linear regression model.

Variable	Reference	Change in WHO-5 score* (point estimator; 95% CI)	P-value
**Gender**	Female	2.71; [0.434; 4.979]	0.0196
**Age (years)**		0.30; [0.2236; 0.3834]	< 0.0001
**Employed**	No	-1.27; [-3.548; 1.0052]	0.2734
**Volunteer**	No	4.70; [1.616; 7.774]	0.0028
**Trauma (yes: 26.6%; no: 73.4%)**	No	-3.76; [-5.9916; -1.5333]	0.001

* The point estimator reflects the change in the WHO-5 score. For example, when “female” is the reference, males reach 2.71 points higher WHO-5 scores than females. Volunteers reach a WHO-5 score that is 4.70 points higher than “professional helper” which is the reference.

**Fig 1:**
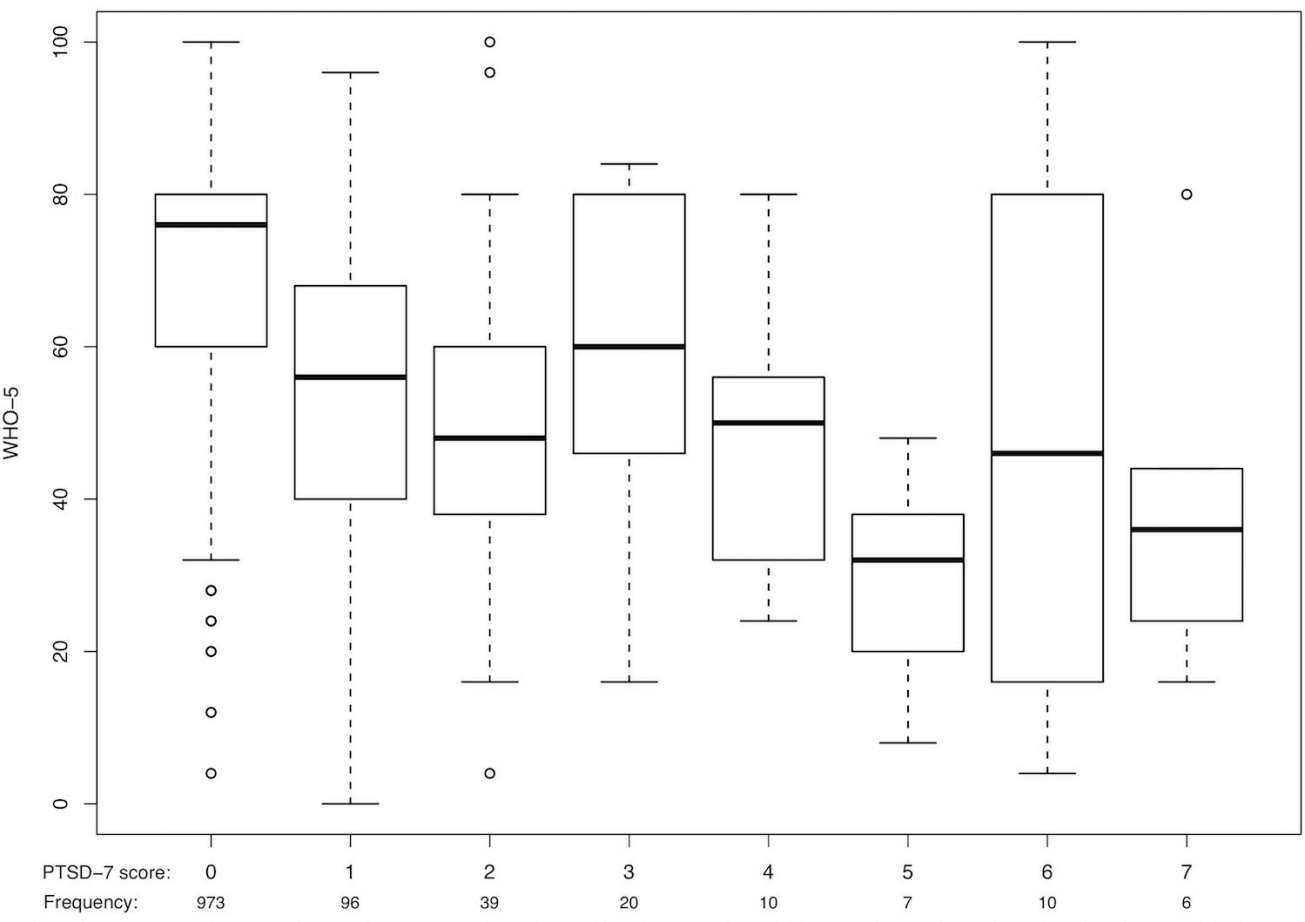
Boxplots showing association between PTSD-7 score and WHO-5 index among refugee helpers.

Furthermore, a correlation was found associating a higher score in PTSD-7 with a lower one in WHO-5 (Fig 1).

982 (57.4%) helpers had experienced a traumatic event in their past or witnessed it during their work in refugee aid. 28,5% of helpers experienced a traumatic event in the past (primary traumatization). 43.3% witnessed a traumatic event during their work in refugee aid (primary traumatization in context of refugee aid) and 23% heard of traumatic situations through other refugee helpers (secondary traumatization; S3 Table). Of all helpers, 33 (2.8%) had a positive PTSD screening in the PTSD-7. Employment status, whether help was on a voluntary or professional basis, gender as well as age did not have an influence on the development of possible PTSD in the investigated sample group (Table 6).

**Table 6:** Influence of helpers’ characteristics on positive PTSD-7 screening in a logistic regression model.

Variable		Frequency	Odds Ratio* (point estimator, 95% CI)	P-value
**Gender**	Female	73.3%	1	
	Male	26.7%	0.98; [0.417; 2.12]	0.96
**Age (years)**		Mean ± SD 52.4 ± 14.3	0.99; [0.963; 1.015]	0.37
**Employment status**	Unemployed or inactive	38.2%	1	
	Employed	61.8%	0.82; [0.375; 1.85]	0.63
**Conditions of activity**	Professional	14.2%	1	
	Volunteer	85.8%	1.66; [0.593; 5.43]	0.36
**Shortage of food or water; poor health without access to medical care; homelessness (Category 1)**	No	59.3%	1	
	Yes	40.7%	1.04; [0.474; 2.34]	0.92
**Serious injury as a result of war or imprisonment/ torture; consequences of rape or sexual abuse (Category 2)**	No	52.9%	1	
	Yes	47.1%	0.77; [0.325; 1.88]	0.57
**Suicide; attempted suicide (Category 3)**	No	82.1%	1	
	Yes	17.9%	2.61; [1.18; 5.72]	0.017
**Forced separation of families; children being orphaned** **(Category 4)**	No	46.8%	1	
	Yes	53.2%	2.19; [0.911; 5.86]	0.095
**Another situation that was very frightening or in which you felt in mortal danger (Category 5)**	No	72.5%	1	
	Yes	27.5%	3.87; [1.76; 8.92]	0.001

*1 = reference

However, by witnessing certain traumatic events like suicide, attempted suicide or other life-threatening situations, helpers’ odds ratio for possible PTSD was significantly higher and of large size (2.61 and 3.87; Table 6). We used the estimated parameters of the logistic regression model to determine the likelihood of PTSD for a fictional helper featuring the most common traits of the group (female, age = 52.4 years, employed, volunteer). Predicted PTSD likelihood was 0.78% with no trauma history. When subjected to a traumatic event such as witnessing suicide or attempted suicide, the likelihood of possible PTSD rose to 2.00%. Being traumatized by a situation that was perceived to be life-threatening led to a PTSD risk of 2.95%. The likelihood of PTSD rose to 7.33% if the individual had undergone both traumatic events.

## Discussion

To our knowledge, we collected the most extensive data set of German refugee helpers to date. We assessed the helpers’ socio-demographics, work-satisfaction, resources and screened for signs of altered well-being, psychological trauma or PTSD.

When it comes to socio-demographic characteristics, most helpers were female, pro-bono workers and middle-aged. Interestingly, the majority of helpers (61.6%) held an academic degree compared to only 17% in the general population [38]. Voluntary helpers made themselves available for a considerable amount of time (9.4 hours (SD: 8.9) per week). Migration background was found in only 10.2% of the helpers. This is in strong contrast to the 22.5% of Germany’s inhabitants who have a migration background as reported by DeStatis, the German Federal Statistical Office in 2016 [39]. Our results are in accordance with previous German surveys that explored voluntary refugee helpers: women engaged themselves more frequently [6, 40]; volunteers had a high level of education [40]; many invested more than 10 hours per week [6] and migration background was less frequent than in the general population [39, 40].

Refugee helpers are exposed to multiple stressors. Adverse working conditions or low work satisfaction can cause chronic stress. Chronic or occupational stress may cause depression and other mental illnesses [12, 41]. Tables 2 and 3 show that helpers were more satisfied with the content of their work than with its conditions or its organization. The lowest satisfaction rates were found in the working conditions subscale (Table 2). When scale means were compared to reference values from the representative sample [24, 27], it could be determined that helpers were actually more satisfied in all of the three work satisfaction subscales (Table 2). This also applies to the work satisfaction expressed by the Kunin faces, as well as to overall work and to overall life satisfaction (Table 3). Two previous studies in German refugee helpers support our results: Karakayali et al. stated that nearly three quarters of the voluntary refugee helpers they examined were fulfilled by their task in spite of their high workload [6]. Sartorius et al. pointed out that a vast majority of the helpers investigated are satisfied with their life in general [40]. This stands in contrast to results from Lopes Cardozo et al. who observed decreased life satisfaction in humanitarian aid workers after their assignment in regions of crisis when compared to before [12]. Obviously, circumstances surrounding humanitarian aid in a crisis region vastly differ from refugee help inside Germany. When participating “at home”, helpers may leave the site of refugee aid in the evening, may receive social support from their families, and in general it can be assumed that they themselves live in a safe environment. This is not the case in refugee camps abroad, where international aid workers are commonly enclosed and virtually confined.

The above findings are consistent with the mean WHO-5 index of 68.2 points (SD: 19.0) in our study population demonstrating high well-being. Looking at the mean WHO-5 scores on the European continent, refugee helpers in Germany compete with the general population in Denmark and Iceland who are renown to be the nations with the highest well-being [26]. The SD of 19.0 points in our group is comparable with values derived from a German representative sample that was assessed in 2004. In the age group of 41–60 years a SD of 19.5 was reported [42].

Nevertheless, we found positive depression screening in 17.3% of the helpers examined. There is indication that trauma predisposes for depression in humanitarian aid workers [12]. This is supported by our results: we found positive WHO-5 screenings in 19.1% of the traumatized and with significant difference, in 12.6% of the non-traumatized helpers (p = 0.0075). In the linear regression model, trauma also had a significant influence on the WHO-5 score (-3.76; p = 0.001, Table 5). A depression rate of just 4.4% was recently observed in a small group of 45 German refugee helpers, although the prevalence of traumatization was higher than in our survey [13]. However, in the German Health Interview and Examination Survey (DEGS1) depressive symptoms were revealed in 8.1% of the representative population-based sample [43]. Thus, positive depression screening in our group of refugee helpers is more common than in the general German population. Both of the mentioned studies above used the Patient Health Questionnaire (PHQ-9) to assess depressive symptoms [13, 43]. Since a high consistency with the WHO-5 in discovering depression has been described before [44], results should be comparable. Yet, when interpreting the positive screening in 17,3% of the helpers it should be remembered that the WHO-5 is a screening test only and that a thorough interview is required for any clinical diagnosis [45] [26].

Further characteristics that had a significant influence on WHO-5 Well-Being Index in the linear regression model were gender, age and volunteer vs. professional refugee helper status (Table 5). Other research groups have already described lower well-being or signs of depression especially in females in the past [10, 43, 46]. Hagh-Shenas et al. reported significantly more depressive symptoms in untrained volunteers engaged in disaster aid than in professional workers [47]. We cannot confirm these results since voluntary refugee helpers in our study had a higher WHO-5 score than professionals (nearly 5 points; p = 0.0028; Table 5).

982 (57.4%) helpers experienced a traumatic event in the past or witnessed it during their work in refugee aid. When compared with findings from other research groups the frequency of traumatic events in the German population varies from 20.9% to 54.6% [48, 49]. Traumatic experiences in our group of refugee helpers are slightly more frequent than in the general population. Fortunately, only 33 (2.8%) helpers had a positive screening for PTSD. Despite the high frequency of traumatic events, the PTSD rate presented in our study is comparable to the one-month prevalence of 2.3% calculated for the general German population [48]. It is known that PTSD may be associated with other mental health illnesses such as depression, anxiety or substance abuse [14, 50, 51]. In concordance, our data shows that a higher PTSD-7 score is associated with a lower WHO-5 score, indicating decreased well-being (Fig 1). It remains unclear why gender did not have an influence on the PTSD-7 screening in our logistic regression model although female gender has been described as a risk factor for PTSD in several studies in the past [52, 53]. Women are known to be especially affected by sexual trauma [52] but in our sample other distressing events (suicide, attempted suicide or other life-threatening situations) led to higher odds for PTSD. So, this fact could have led to a more equalized rate of PTSD in both genders in our sample.

Naturally, there are some limitations to our study. Firstly, any voluntary and anonymous questionnaire may be plagued by selection bias. Despite its extensive scope, we cannot exclude the possibility that our sample may not be fully representative for the entire community of refugee helpers in Germany. For example, those who have left refugee work for health issues are not represented in our work-force cohort. This “healthy-worker-effect” has long been described in literature and may lead to a generally better health status within a study population when compared to the general population [54].

Secondly–with the majority of the study population being volunteers–we had to exclude some items from the Neuberger questionnaire from our analysis, since they were designed for the use with professional, paid workers and questions about pay and career etc. had no bearing in our context.

Finally, we performed a questionnaire survey and not structured clinical interviews to evaluate depression, traumatization or PTSD. Consequently, the occurrence of a positive screening may be suggestive of these conditions, but cannot be seen as equivalent to a clinical diagnosis.

## Conclusions

With this study we have presented data on German refugee helpers’ socio-demographics, work satisfaction and mental health. The typical helper is female, middle-aged and has received higher education. Personal investment is high, but helpers wish for more support in the form of financial means and training for their tasks. Helpers’ satisfaction with the content of their work is above average, and their WHO-5 well-being scores are high. In spite of a high prevalence of psychological trauma, there is an infrequent occurrence of PTSD among helpers.

Despite these positive results, prospective and longitudinal research on the subject of mental health and well-being of helpers is necessary as part of a more comprehensive prevention and care strategy. Refugee workers provide invaluable services to both incoming migrants and receiving communities. Their commitment and dedication need to be supported and sustained since migrant flows are predicted to increase rather than decrease in the foreseeable future. We suggest that helpers should be screened regularly with regard to work satisfaction, well-being and their mental health. Potential future deteriorations in comparison with our base-line findings should prompt swift efforts for improvements from politicians and society, in order to enable helpers to help. Finally, helpers should be instructed about depression, psychological traumatization, PTSD and ways of seeking help for themselves.

## Supporting information

S1 Table Refugee helpers’ profession and hierarchical level. Relative frequencies calculated for non-missing answers (N).
(DOCX)

S2 Table Refugee helpers’ organization. Relative frequencies calculated for non-missing answers (N).
(DOCX)

S3 Table Relative frequencies of potential traumatic events in refugee helpers calculated for non-missing answers (N). (DOCX)
