# Supplementary material for: Do good, stay well. Well-being and work satisfaction among German refugee helpers: A national cross-sectional study
Source: PLoS One. 2018 Dec 26;13(12):e0209697. doi: 10.1371/journal.pone.0209697 (PMC6306198; doi:10.1371/journal.pone.0209697)
Supplement: S1 Table — Relative frequencies calculated for non-missing answers (N). (DOCX) [file pone.0209697.s001.docx]

**S1 Table. Refugee helpers’ profession and hierarchical level. Relative frequencies calculated for non-missing answers (N).**

| Profession | **Frequency** | **N** |
| --- | --- | --- |
| Medical field | 11.4% | 1697 |
| Non-medical field | 76.6% | 1696 |
| Hierarchical level |  |  |
| Management | 33.9% | 1632 |
| Employee | 66.1% | 1632 |
