# Supplementary material for: Do good, stay well. Well-being and work satisfaction among German refugee helpers: A national cross-sectional study
Source: PLoS One. 2018 Dec 26;13(12):e0209697. doi: 10.1371/journal.pone.0209697 (PMC6306198; doi:10.1371/journal.pone.0209697)
Supplement: S2 Table — Relative frequencies calculated for non-missing answers (N). (DOCX) [file pone.0209697.s002.docx]

**S2 Table. Refugee helpers’ organization. Relative frequencies calculated for non-missing answers (N).**

| Organization | **Frequency** | **N** |
| --- | --- | --- |
| Welfare organization, church welfare organization (Workers'  Welfare Association, German Red Cross, Caritas, Diaconia, ...) | 17.7% | 1702 |
| Relief organization (Bavarian Red Cross, Order of St. John,  Malteser Hilfsdienst, Workers' Samaritan Federation, ...) | 5.1% | 1702 |
| Non-governmental organization (Pro Asyl ...) | 12.5% | 1702 |
| Self-organized initiative | 63.1% | 1702 |
| Commercial refugee aid | 15.9% | 1702 |
| Government agency (Federal Office for Migration and Refugees …) | 3.4% | 496 |
| Other | 11.6% | 1702 |
