# Supplementary material for: Do good, stay well. Well-being and work satisfaction among German refugee helpers: A national cross-sectional study
Source: PLoS One. 2018 Dec 26;13(12):e0209697. doi: 10.1371/journal.pone.0209697 (PMC6306198; doi:10.1371/journal.pone.0209697)
Supplement: S3 Table — (DOCX) [file pone.0209697.s003.docx]

**S3 Table. Relative frequencies of potential traumatic events in refugee helpers calculated for non-missing answers (N).**

|  | Experienced oneself in the past  (primary traumatization) | Witnessed during work in refugee aid  (primary traumatization) | Heard of it through other refugee helpers  (secondary traumatization) | None of the above | N |
| --- | --- | --- | --- | --- | --- |
| Category 1 |  |  |  |  |  |
| Shortage of food or water | 4% | 7.1% | 39.2% | 49.7% | 1338 |
| Poor health without access to medical care | 5.9% | 24.2% | 40.1% | 29.8% | 1338 |
| Homelessness | 2.9% | 16.8% | 37.3% | 43.0% | 1334 |
| Category 2 |  |  |  |  |  |
| Serious injury as a result of war or imprisonment/ torture | 4.8% | 34.7% | 41.5% | 19.1% | 1346 |
| Consequences of rape or sexual abuse | 4.9% | 13.6% | 41.4% | 40.2% | 1332 |
| Category 3 |  |  |  |  |  |
| Suicide | 2.8% | 4.4% | 37.2% | 55.6% | 1334 |
| Attempted suicide | 3.4% | 11.1% | 38.8% | 46.7% | 1328 |
| Category 4 |  |  |  |  |  |
| Forced separation of families | 12% | 35.1% | 37.3% | 15.6% | 1350 |
| Children being orphaned | 4.6% | 18.7% | 42.5% | 34.2% | 1329 |
| Category 5 |  |  |  |  |  |
| Another situation that was very frightening or in which you felt in mortal danger | 15.8% | 11% | 20.9% | 52.4% | 1288 |
| All categories | 28.5 % | 43.3 % | 23 % | 5.1 % | 1367 |
